# Multiple comorbid sleep disorders adversely affect quality of life in Parkinson’s disease patients

**DOI:** 10.1038/s41531-020-00126-x

**Published:** 2020-09-15

**Authors:** Yu Zhang, Jia hao Zhao, Dong ya Huang, Wei Chen, Can xing Yuan, Li rong Jin, Yu hui Wang, Ling jing Jin, Lei Lu, Xiao ping Wang, Chang de Wang, Xiao hui Zhao, Xi Zhang, Wen tao Li, Zhen guo Liu

**Affiliations:** 1 Department of Neurology, Xinhua Hospital Affiliated to Shanghai Jiao Tong University School of Medicine, Shanghai, China; 2 Department of Neurology, East Hospital, Tongji University School of Medicine, Shanghai, China; 3 Department of Neurology, Shanghai Ninth People’s Hospital, Shanghai Jiao Tong University School of Medicine, Shanghai, China; 4 Department of Neurology, Longhua Hospital, Shanghai University of Traditional Chinese Medicine, Shanghai, China; 5 Department of Neurology, Zhongshan Hospital, Fudan University, Shanghai, China; 6 Department of Neurology, Shanghai Punan Hospital, Shanghai, China; 7 Department of Neurology, Tongji Hospital, Tongji University School of Medicine, Shanghai, China; 8 Department of Neurology, Shanghai Jiao Tong University Affiliated Sixth People’s Hospital, Shanghai, China; 9 Department of Neurology, Tongren Hospital, Shanghai Jiao Tong University School of Medicine, Shanghai, China; 10 Department of Neurology, Shanghai TCM-Integrated Hospital Affiliated to Shanghai University of Traditional Chinese Medicine, Shanghai, China; 11 Department of Neurology, Shanghai Pudong New Area Hospital, Shanghai, China; 12 Clinical Research Unit, Xinhua Hospital Affiliated to Shanghai Jiao Tong University School of Medicine, Shanghai, China; 13 Department of Neurology, Shanghai Municipal Hospital of Traditional Chinese Medicine, Shanghai University of Traditional Chinese Medicine, Shanghai, China

**Keywords:** Parkinson's disease, Parkinson's disease

## Abstract

Sleep disorders are common non-motor symptoms in patients with Parkinson’s disease (PD). The characteristics and impact of multiple comorbid sleep disorders remain to be elucidated. Our goal was to investigate the characteristics of various sleep disorder comorbidities, and their association with motor complications and the impact on the quality of life in PD patients. In this multicenter, observational, cross-sectional study, data concerning the clinical characteristics of complicated sleep disorders were collected from PD patients treated at 40 different hospitals in Shanghai. Sleep disorders were evaluated using the PD Sleep Scale-2, Epworth Sleepiness Scale, Rapid Eye Movement Sleep Behavior Disorder Questionnaire-Hong Kong, and the International Restless Legs Scale. Among the 1006 subjects evaluated, 77.53% exhibited signs of sleep disorders, and most had multiple sleep disorders (*n* = 502, 49.9%). A smaller percentage of patients with sleep disorders had a single disorder (*n* = 278, 27.6%). Furthermore, an increased number of sleep disorders, including nighttime problems, excessive daytime sleepiness, rapid eye movement sleep behavior disorder, and restless legs syndrome was a significant contributor to a poor quality of life (*β* = 4.33, CI: 3.33–5.33, *P* for trend <0.001), even when controlling for multiple factors. Moreover, motor complications partially mediated this relationship (indirect effect: *β* = 0.355, 95% boot CI: 0.134, 0.652).Our study showed that a large proportion of PD patients suffer from multiple comorbid sleep disorders, which greatly decreases the quality of life in PD patients and is partially mediated by motor complications.

## Introduction

Parkinson’s disease (PD) is a movement disorder. Sleep disorders, such as nighttime problems, excessive daytime sleepiness (EDS), rapid eye movement sleep behavior disorder (RBD), and restless legs syndrome (RLS) are commonly reported in patients with PD^1–3^. The pathophysiology of sleep disorders associated with dopaminergic and non-dopaminergic mechanisms during the course of PD and the sleep disorder type impart different effects on PD patients^4^. RLS is often associated with constipation in PD patients^5^. RBD is associated with longer disease duration, motor fluctuations, psychiatric comorbidities, and higher doses of levodopa in PD patients^6,7^. RBD can be a prodromal symptom of PD and can be used for diagnosis of PD at an early stage^1,2,7,8^. Furthermore, sleep disorders are observed throughout the course of PD. Nighttime problems and EDS significantly reduce the quality of life in patients with advanced PD, and require prompt recognition and intervention^1,2,7^. Clinical subtypes of sleep disorders may even constitute distinct phenotypes of PD.

Many previous studies have focused on the effects of a single type of sleep disorder on PD^9–13^. It is worth noting that PD patients may have multiple comorbid sleep disorders that require attention. However, few studies have investigated the clinical distribution and characteristics of complicated sleep disorders in PD patients. Furthermore, the association between an increased number of sleep disorders and the quality of life in PD patients, along with identification of the factors that influence this relationship, is largely unknown. Although an accurate sleep disorder diagnosis should be made using polysomnography, devising a screening tool using validated questionnaires that are reliable and diagnostically accurate would be a big step forward in early identification^14–16^. Therefore, large-scale studies on sleep disorders in patients with PD in China are needed to devise tools for faster identification of issues, which can then be better characterized using current methodologies (e.g., polysomnography).

Sleep disorders could be observed throughout the course of PD, while motor complications typically emerge only in advanced cases of disease. Any one, or a combination of sleep disorders and/or movement disorders negatively impacts quality of life. Whether motor complications influence or regulate the impact of sleep disorders on quality of life is unknown. Exploring the effects of motor complications on this relationship may provide important implications for early interventions designed to enrich and prolong quality of life.

In this multicenter, observational, cross-sectional study, we aimed to investigate the clinical characteristics of complicated sleep disorders in patients with PD, their association with motor complications and the overall impact on quality of life.

## Results

### Study population and the distribution of different types of sleep disorders

A total of 1006 patients with PD (mean age = 69.95 ± 8.41 years old, 577 males) were enrolled in the study. The mean PD disease duration was 5.54 ± 4.58 years. The mean modified Hoehn–Yahr (HY) stage was 2.17 ± 0.84 (stage 1–1.5, *n* = 296; stage 2–2.5, *n* = 471; and stage ≥3, *n* = 223). The Unified Parkinson’s Disease Rating Scale (UPDRS) parts I, II, III, and IV scores were 3.06 ± 2.82, 12.41 ± 8.44, 25.50 ± 15.51, and 3.20 ± 3.66, respectively. Of the total cohort, 920 patients (91.50%) were taking levodopa, 563 (56.00%) were taking dopamine agonists, 39 (3.90%) were taking a MAO-B inhibitor, and 173 (17.20%) were taking a COMT inhibitor. Complete demographic data for the entire cohort are shown in Table 1.

**Table 1:** Characteristics of study participants classified by sleep disorders.

	Total	Nighttime problems			EDS			pRBD			RLS		
		Yes	No	*P-*value	Yes	No	*P-*value	Yes	No	*P-*value	Yes	No	*P-*value
*N*	1006	421	585		254	752		472	534		501	505	
Sex (male, %)	57.4	53.90	59.80	0.062	61.40	56.00	0.13	56.60	58.10	0.635	55.10	59.60	0.148
Age (yr)	69.95 ± 8.41	70.61 ± 8.56	69.47 ± 8.28	**0.034**	71.01 ± 8.61	69.59 ± 8.32	**0.019**	69.88 ± 7.92	70.00 ± 8.83	0.825	70.01 ± 8.66	69.89 ± 8.17	0.819
Family history of PD (%)	7.70	6.20	8.70	0.138	7.50	7.70	0.892	7.20	8.10	0.608	5.80	9.50	**0.027**
Disease duration (yr)	5.54 ± 4.58	6.13 ± 4.89	5.11 ± 4.30	**<0.001**	6.48 ± 4.94	5.22 ± 4.41	**<0.001**	5.94 ± 4.79	5.18 ± 4.37	**0.008**	6.04 ± 4.96	5.03 ± 4.11	**<0.001**
Initial presentation of motor symptoms (%)				0.471			0.424			0.632			0.262
Tremor	58.40	58.40	58.50		56.70	59.00		58.10	58.80		56.50	60.40	
Rigid	10.60	10.60	9.90		10.20	10.80		9.50	11.60		12.40	8.90	
Bradykinesia	21.40	21.40	22.70		20.90	21.50		22.20	20.60		21.00	21.80	
Other	9.50	9.50	8.90		12.20	8.60		10.20	9.00		9.50	8.90	
Modified HY stage (%)				**<0.001**			**<0.001**			**<0.001**			**0.018**
1–1.5	29.40	19.30	37.40		18.10	33.90		23.40	35.70		25.80	33.90	
2–2.5	46.80	51.00	45.20		47.00	47.80		52.10	43.50		51.00	44.20	
≥3	22.20	29.80	17.40		34.90	18.40		24.50	20.80		23.20	21.90	
UPDRS total	44.23 ± 26.69	56.7 ± 28.7	35.27 ± 21.01	**<0.001**	59.24 ± 32.53	39.18 ± 22.27	**<0.001**	49.34 ± 27.49	39.71 ± 25.14	**<0.001**	51.18 ± 29.02	37.35 ± 22.15	**<0.001**
UPDRS part I	3.06 ± 2.82	4.28 ± 2.92	2.19 ± 2.39	**<0.001**	4.50 ± 3.15	2.58 ± 2.53	**<0.001**	3.82 ± 2.86	2.40 ± 2.62	**<0.001**	3.67 ± 3.13	2.46 ± 2.34	**<0.001**
UPDRS part II	12.41 ± 8.44	16.21 ± 8.87	9.69 ± 6.94	**<0.001**	16.81 ± 10.07	10.94 ± 7.24	**<0.001**	13.88 ± 8.72	11.12 ± 7.97	**<0.001**	14.67 ± 9.05	10.18 ± 7.12	**<0.001**
UPDRS part III	25.50 ± 15.51	31.07 ± 16.83	21.49 ± 13.1	**<0.001**	32.76 ± 18.27	23.06 ± 13.63	**<0.001**	27.54 ± 15.6	23.7 ± 15.22	**<0.001**	28.55 ± 16.46	22.49 ± 13.88	**<0.001**
UPDRS part IV (32–39)	2.30 ± 3.18	3.67 ± 3.77	1.32 ± 2.22	**<0.001**	3.84 ± 4.06	1.78 ± 2.63	**<0.001**	2.84 ± 3.44	1.83 ± 2.85	**<0.001**	3.23 ± 3.79	1.38 ± 2.05	**<0.001**
PDQ-39 score	36.24 ± 28.60	51.82 ± 30.16	25.45 ± 23.49	**<0.001**	55.28 ± 32.16	30.12 ± 25.61	**<0.001**	43.86 ± 32.3	29.94 ± 25.04	**<0.001**	45.6 ± 30	27.45 ± 26.04	**<0.001**
NMSS score	49.95 ± 44.03	74.98 ± 50.38	31.93 ± 27.08	**<0.001**	80.51 ± 56.06	39.65 ± 33.39	**<0.001**	62.74 ± 48.73	38.6 ± 35.78	**<0.001**	60.29 ± 49.07	39.69 ± 35.56	**<0.001**
MMSE score	25.69 ± 4.90	24.27 ± 5.4	26.7 ± 4.218	**<0.001**	23.7 ± 5.87	26.37 ± 4.32	**<0.001**	25.25 ± 5.02	26.09 ± 4.75	**0.006**	24.78 ± 5.46	26.6 ± 4.07	**<0.001**
L-dopa medication (%)	91.50	94.50	89.20	**0.003**	92.10	91.20	0.656	94.10	89.10	**0.005**	93.00	89.90	0.077
DAs medication (%)	56.00	52.00	58.80	**0.032**	52.40	57.20	0.181	58.80	53.70	0.132	49.90	62.00	**<0.001**
Levodopa equivalent daily dose (mg/day)	444.71 ± 272.26	491.1 ± 265.7	411.3 ± 272.2	**<0.001**	501.45 ± 299.21	425.54 ± 259.96	**<0.001**	476.38 ± 261.23	416.71 ± 278.91	**0.001**	461 ± 269.85	428.54 ± 273.94	0.059
Hallucination (%)	12.60	19.20	7.90	**<0.001**	23.60	8.90	**<0.001**	19.50	6.60	**<0.001**	14.20	11.10	0.141
Total sleep time (h)	5.88 ± 1.88	5.183 ± 1.717	6.378 ± 1.829	**<0.001**	5.57 ± 1.85	5.98 ± 1.88	**0.002**	5.57 ± 1.85	6.15 ± 1.87	**<0.001**	5.64 ± 1.84	6.11 ± 1.89	**<0.001**
Number of sleep interruptions (per night)	2.04 ± 1.44	2.583 ± 1.536	1.645 ± 1.231	**<0.001**	2.34 ± 1.41	1.94 ± 1.44	**<0.001**	2.26 ± 1.48	1.84 ± 1.38	**<0.001**	2.12 ± 1.43	1.96 ± 1.45	0.08

*N* number, *EDS* excessive daytime sleepiness, *pRBD* probable rapid eye movement sleep behavior disorder, *RLS* restless legs syndrome, *modified HY stage* modified Hoehn and Yahr stage, *UPDRS* Unified Parkinson’s Disease Rating Scale, *NMSS* non-motor symptom scale, *PDQ-39* Parkinson’s Disease Questionnaire-39, *MMSE* Mini-Mental State Examination, *DA* dopamine agonist. Statistically signficant *P*-values (*P* < 0.05) are highlighted in bold.

**Fig. 1:**
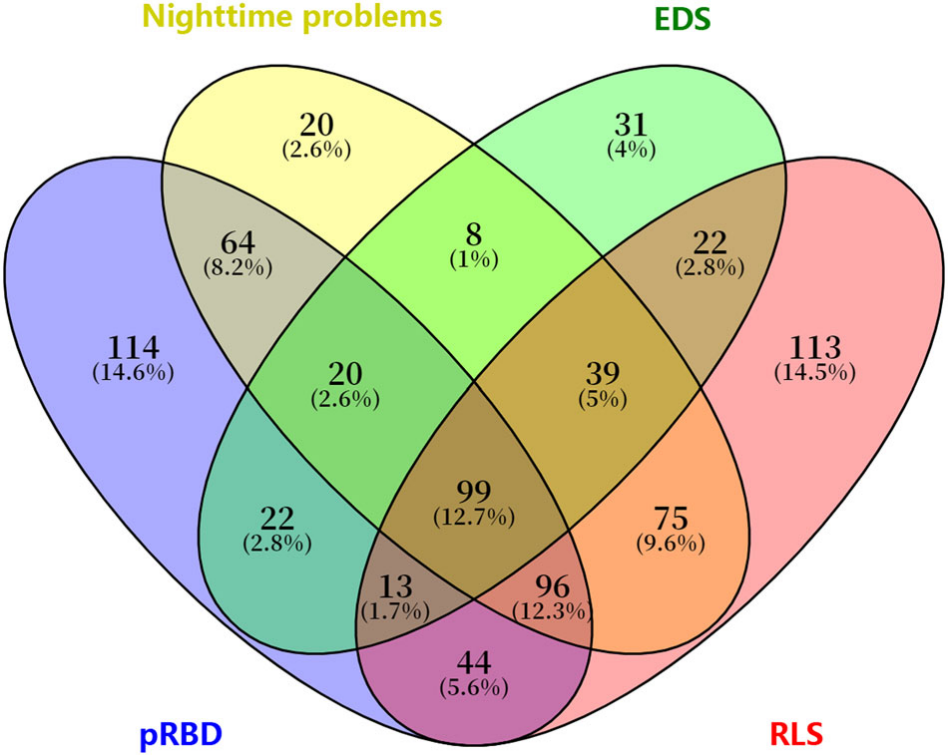
Prevalence and overlap of sleep disorders in patients with PD. EDS excessive daytime sleepiness, pRBD probable rapid eye movement sleep behavior disorder, RLS restless legs syndrome.

Regarding the sleep assessments, the prevalence of sleep disorders was 41.80% (421/1006) for nighttime problems, 25.20% (254/1006) for EDS, 46.90% (472/1006) for probable RBD (pRBD), and 49.80% (501/1006) for RLS. There was a significant overlap of various sleep disorders in patients with PD (Fig. 1). Accordingly, 226 (22.5%) of the patients had no sleep disorder, 278 (27.6%) had only one type of sleep disorder, and 502 (49.9%) had ≥2 sleep disorders (Fig. 1).

### Factors associated with different types of sleep disorders

The characteristics of the patients with PD according to sleep disorder type (nighttime problems, EDS, pRBD, and RLS) are shown in Table 1. Compared with the patients without sleep disorders, the nighttime problems, EDS, pRBD, and RLS groups had longer disease durations, higher UPDRS scores (total, parts I, II, III, and IV) and non-motor symptom scale (NMSS) scores, worse cognitive function, and shorter total sleep times. A slight increase in age was associated with nighttime problems and EDS. We found no specific pattern of initial presentation of motor symptoms that was significantly related to the presence of nighttime problems, EDS, pRBD, or RLS.

Logistic regression analysis of nighttime problems, EDS, pRBD, and RLS using a likelihood ratio forward selection showed that UPDRS part IV (32–39) scores (OR = 1.186, CI: 1.118–1.259, *P* < 0.001) and NMSS scores (OR = 1.030, CI: 1.024–1.035, *P* < 0.001) were contributing factors to occurrence of nighttime problems. UPDRS part IV (32–39) scores (OR = 1.090, CI: 1.035–1.147, *P* = 0.001), NMSS scores (OR = 1.018, CI: 1.014–1.022, *P* < 0.001), and hallucinations (OR = 1.595, CI: 1.016–2.504, *P* = 0.043) were contributing factors for EDS. Age (OR = 0.983, CI: 0.968–0.999, *P* = 0.038), NMSS scores (OR = 1.014, CI: 1.010–1.018, *P* < 0.001, and hallucinations (OR = 2.367, CI: 1.513–3.703, *P* < 0.001) were contributing factors for pRBD. Age (OR = 0.979, CI: 0.963–0.997, *P* = 0.019), disease duration (OR = 1.045, CI: 1.010–1.081, *P* = 0.012), modified HY stage (OR = 0.723, CI: 0.571–0.914, *P* = 0.007), UPDRS parts II scores (OR = 1.062, CI: 1.036–1.088, *P* < 0.001), UPDRS parts IV (32–39) scores (OR = 1.182, CI: 1.115–1.252, *P* < 0.001), and dopamine agonist medication use (OR = 0.571, CI: 0.429–0.761, *P* < 0.001) were contributing factors for RLS (Supplementary Table 1).

### Clinical characteristics and factors associated with different numbers of sleep disorders

PD patients were categorized into the following five groups according to the number of sleep disorders ranked 0–4 as follows: zero sleep disorders (*n* = 226, 22.50%), one type of sleep disorder (*n* = 278, 27.60%), two types of sleep disorders (*n* = 235, 23.40%), three types of sleep disorders (*n* = 168, 16.70%), and four types of sleep disorders (*n* = 99, 9.80%) (Table 2). The clinical characteristics of patients according to the number of sleep disorders are shown in Table 2. Among all PD patients, those with a greater number of sleep disorders were more likely to have a longer disease duration, higher modified HY stage, greater UPDRS score (total, III and IV), worse cognitive function, and a greater number of non-motor symptoms. They were also more likely to be currently on L-dopa medication and to have a higher levodopa equivalent daily doses (LEDDs). However, they were less likely to be taking dopamine agonists. Compared to patients who suffered only one type of sleep disorder, those with multiple sleep disorders had a worse quality of life and more severe motor complications. These associations were slightly attenuated after controlling for disease duration, disease progression, motor symptoms, non-motor symptoms, and dopaminergic medications (Table 3). The multivariable regression coefficients (95% confidence intervals (95% CIs)) of quality of life in reference to no sleep disorders were 1.79 (−1.17 to 4.76) for one type of sleep disorders, 3.89 (0.71–7.08) for two types of sleep disorders, 14.63 (10.96–18.31) for three types of sleep disorders, and 16.04 (11.43–20.65) for four types of sleep disorders; this trend was statistically significant (*P* for trend <0.001). Furthermore, we found the same trend in the severity of motor complications and the number of sleep disorders (*P* for trend <0.001). The multivariable regression coefficients (95% CIs) for quality of life in reference to no sleep disorders were 7.88 (1.29–14.47) for the nighttime problems + EDS + RLS group, 10.48 (1.72–19.24) for the nighttime problems + EDS + pRBD group, 14.67 (9.93–19.40) for the nighttime problems + pRBD + RLS group, and 12.56 (2.83–22.30) for the EDS + pRBD + RLS group (Table 3).

**Table 2:** Clinical characteristics of patients according to number of sleep disorders.

	Number of sleep disorders					*P*-value for trend
	None	One	Two	Three	Four	
*N*	226	278	235	168	99	
Sex (male, %)	58.80	59.70	55.30	54.80	56.60	0.334
Age (yr)	70.29 ± 9.19	68.72 ± 7.47	69.85 ± 8.20	71.11 ± 8.43	70.84 ± 9.18	0.102
Disease duration (yr)	4.26 ± 3.79	5.50 ± 4.68	5.91 ± 4.15	5.95 ± 4.77	6.96 ± 5.86	**<0.001**
Modified HY stage (%)						**<0.001**
1–1.5	41.60	38.00	23.10	20.20	12.50	
2–2.5	39.80	44.60	57.70	44.80	54.20	
≥3	18.60	17.40	19.20	35.00	33.30	
UPDRS total	31.92 ± 19.99	36.91 ± 21.38	45.07 ± 22.42	55.71 ± 28.61	71.52 ± 31.99	**<0.001**
UPDRS part III	19.95 ± 13.21	22.07 ± 12.95	26.4 ± 14.04	30.27 ± 17.28	37.64 ± 17.86	**<0.001**
UPDRS part IV (32–35)	0.32 ± 0.86	0.70 ± 1.52	0.91 ± 1.60	1.71 ± 2.37	3.38 ± 2.74	**<0.001**
UPDRS part IV (36–39)	0.75 ± 1.17	0.84 ± 1.35	1.16 ± 1.53	1.63 ± 1.60	2.69 ± 1.81	**<0.001**
PDQ-39 score	21.27 ± 21.46	26.77 ± 25.03	35.38 ± 22.07	54.88 ± 29.72	69.83 ± 30.24	**<0.001**
NMSS score	25.53 ± 25.17	35.21 ± 27.7	52 ± 36.45	72.96 ± 44.92	103.12 ± 62.26	**<0.001**
MMSE score	26.94 ± 3.62	26.65 ± 4.74	25.7 ± 4.29	24.81 ± 5.34	21.7 ± 5.96	**<0.001**
L-dopa medication (%)	87.60	89.60	94.00	93.50	96.00	**0.002**
DAs medication (%)	56.60	61.90	56.20	48.80	49.50	**0.026**
Levodopa equivalent daily dose (mg/day)	384.92 ± 280.81	423.96 ± 260.68	461.9 ± 247.59	481.63 ± 296.82	535.98 ± 263.84	**<0.001**
Total sleep time (h)	6.32 ± 1.89	6.36 ± 1.93	5.76 ± 1.58	5.18 ± 1.78	4.96 ± 1.79	**<0.001**
Number of sleep interruptions (per night)	1.60 ± 1.33	1.78 ± 1.32	2.23 ± 1.33	2.50 ± 1.68	2.55 ± 1.39	**<0.001**

Number of sleep disorders (0–4; nighttime problems, EDS, pRBD, and RLS).

*N* number, *modified HY stage* modified Hoehn and Yahr stage, *UPDRS* Unified Parkinson’s Disease Rating Scale, *NMSS* non-motor symptom scale, *PDQ-39* Parkinson’s Disease Questionnaire-39, *MMSE* Mini-Mental State Examination, *DA* Dopamine agonist. Statistically signficant *P*-values (*P* < 0.05) are highlighted in bold.

**Table 3:** Association between quality of life, motor complications, and multiple comorbid sleep disorders.

Variable	PDQ-39 score *β* (95% CI)^a^			UPDRS part IV (32–39) *β* (95% CI)^a^		
	Model 1	Model 2	Model 3	Model 1	Model 2	Model 3
Number of sleep disorders						
None	1.00 (reference)	1.00 (reference)	1.00 (reference)	1.00 (reference)	1.00 (reference)	1.00 (reference)
One	5.83 (1.73–9.92)	1.80 (−1.18–4.77)	1.79 (−1.17–4.76)	10.77 (4.30–17.24)	0.12 (−0.34–0.58)	0.11 (−0.35–0.57)
Two	14.41 (10.16–18.66)	3.75 (0.59–6.91)	3.89 (0.71–7.08)	26.93 (20.2–33.63)	0.19 (−0.30–0.68)	0.18 (−0.32–0.67)
Three	32.73 (28.08–37.38)	15.40 (11.79–19.01)	14.63 (10.96–18.31)	46.42 (39.07–53.77)	1.02 (0.46–1.58)	0.92 (0.35–1.49)
Four	48.16 (42.67–53.65)	16.16 (11.60–20.72)	16.04 (11.43–20.65)	77.23 (68.57–85.89)	2.70 (2.00–3.40)	2.68 (1.96–3.39)
*P* for trend	**<0.001**	**<0.001**	**<0.001**	**<0.001**	**<0.001**	**<0.001**
Increase per disorder	11.72 (10.57–12.87)	4.47 (3.50–5.45)	4.33 (3.33–5.33)	1.03 (0.89–1.18)	0.51 (0.36–0.66)	0.49 (0.34–0.65)

Number of sleep disorders (0–4; nighttime problems, EDS, pRBD, and RLS).

*β* unstandardized regression coefficient, *95% CI* 95% confidence interval, *UPDRS* Unified Parkinson’s Disease Rating Scale, *PDQ-39* Parkinson’s Disease Questionnaire-39.

^a^*β*s and 95% CIs were calculated with the use of multivariate linear regression. Model 1 was adjusted for sex and age. Model 2 additionally was adjusted for disease duration, modified HY stage, UPDRS part III, NMSS, and MMSE. Model 3 was further adjusted for L-dopa medication status, DA medication status, levodopa equivalent daily dose, total sleep time, and number of sleep interruptions. Statistically signficant *P*-values (*P* < 0.05) are highlighted in bold.

**Fig. 2:**
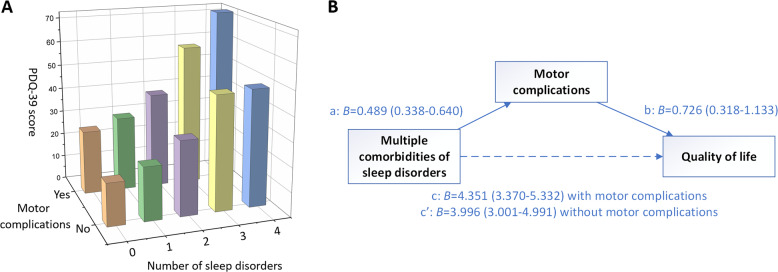
Motor complications partially mediated the effect of multiple comorbid sleep disorders on quality of life. **a** Stratification analyses of positive associations between an increased number of sleep disorders and quality of life by motor complications status. Number of sleep disorders (0–4; nighttime problems, EDS, pRBD, and RLS). **b** Motor complications as a mediator between an increased number of sleep disorders and quality of life. All associations were adjusted for sex, age, disease duration, modified HY stage, UPDRS part III, NMSS, MMSE, L-dopa medication status, dopamine agonist medication status, and levodopa equivalent daily dose.

We further stratified the analyses by motor complication status. Positive associations between an increased number of sleep disorders and quality of life were found both among patients with and without motor complications (*β* = 4.467, 3.312; *P* for trend <0.001, <0.001; Fig. 2). However, the interaction with motor complication status was not statistically significant (*P* for interaction = 0.759).

### The mediation analyses of the associations between multiple comorbid sleep disorders and quality of life

When the motor complications score was entered as a mediator of the number of sleep disorders and PDQ-39, the total effect was significant (*β* = 4.351, CI: 3.370–5.332, *P* < 0.001), the indirect effect was significant (*β* = 0.355, 95% boot CI: 0.134, 0.652), and the direct effect remained significant (*β* = 3.996, CI: 3.001–4.991, *P* < 0.001). Therefore, we can infer that motor complications partially mediated the effect of multiple comorbid sleep disorders on quality of life (Fig. 2).

## Discussion

In this large-scale, multicenter, cross-sectional study, we found that PD patients have a significantly higher prevalence of sleep disorders. Our results showed that the prevalence of sleep disorders (77.5%) in patients with PD is higher than previously reported in China^7,17^. In addition, there were 502 patients (49.9%) with multiple comorbid sleep disorders, while only 278 patients (27.6%) had only one type of sleep disorder. The prevalence of nighttime problems with pRBD and RLS was 9.5%, which is significantly higher than the prevalence of any other combination of three of sleep disorders. Nighttime problems combined with RLS (7.5%), and nighttime problems combined with pRBD (6.4%) were significantly more prevalent than any other combination of two disorders. In contrast, EDS alone, EDS combined with nighttime problems, EDS combined with RLS, or EDS combined with pRBD are not common. These results reveal that the proportion of patients with multiple comorbid sleep disorders is much greater than the proportion with only one sleep disorder. In addition to one specific type of sleep disorder, multiple comorbidities should also be considered when assessing patients with PD who have sleep disorder complaints. Comprehensive evaluation and therapy will achieve a better curative effect.

At the same time, we found that nighttime problems, EDS, pRBD, and RLS are all accompanied by cognitive decline, which was confirmed by other studies^17–19^. Compared with participants without RLS, those with RLS have a higher rate of a family history of PD. However, nighttime problems, EDS, and pRBD have no correlation with a family history of PD, which was consistent with other studies^20^. RLS results in fewer sleep interruptions, while nighttime problems, EDS, and pRBD are typically accompanied by frequent sleep interruptions. As a clinical symptom, the prevalence of hallucinations among the different types of sleep disorders (nighttime problems, EDS, pRBD, and RLS) is described in Table 1. Our results indicated that hallucinations were more likely to occur in PD patients with EDS and RLS than those without. It is worth noting that dopamine agonists were less frequently prescribed or used in the PD group with EDS and RLS than in the PD group without EDS and RLS. Therefore, it is difficult to infer whether the appearance of hallucinations is related to the use of dopamine agonists. According to the UPDRS part I and NMSS scores, PD patients with sleep disorders (nighttime problems, EDS, pRBD, or RLS) usually have a greater number of more severe non-motor symptoms. Patients with nighttime problems, pRBD, and RLS have an inferior quality of life compared to patients with any other combination of three comorbid sleep conditions. Therefore, nighttime problems are more likely to coincide with RLS and pRBD, and these three types of sleep disorders may play some role in pathogenesis because they have similar clinical characteristics and have a more significant impact on quality of life. Alternatively, it is possible that nighttime problems may be the result of RLS and pRBD. In contrast, EDS is relatively independent, and its pathogenesis may be different from the other disorders.

Our study revealed that patients with a greater number of sleep disorders were less likely to be taking dopamine agonists (*P* = 0.026). The dopamine agonists used in the China are pramipexole and piribedil. We performed a subgroup analysis, and there was no negative correlation between the number of sleep disorders and the use of pramipexole or piribedil. Therefore, dopamine agonist is not likely the cause of sleep disorders, and our results represent statistically significant correlations. Due to the limitations of observational cross-sectional studies, we are unable to make further inferences. Future longitudinal studies would enhance our interpretations. Furthermore, the analyses revealed that the number of sleep disorders was an independent and significant contributor to quality of life in PD patients, even after we adjusted for multiple factors. Our findings demonstrated that an increased number of sleep disorders had a significant negative impact on quality of life.

Through regression analysis, we found that motor complications were related to nighttime problems, EDS, and RLS, while both sleep disorders and motor complications were necessary for significant deteriorations in quality of life. These findings suggested that there may be interactions between sleep disorders and motor complications. Notably, rather than examining the direct relationship between motor complications and quality of life in PD, we investigated the interaction effect between motor complications and multiple comorbid sleep disorders. Our findings show there was no significant interactive effect between motor complications and multiple sleep disorder comorbidities on quality of life in PD patients (*P* for interaction = 0.759). Nevertheless, the impact of motor complications on quality of life shows a mediating effect according to our study. Motor complications act as a mediator of the association between multiple comorbid sleep disorders and quality of life. Controlling for the severity of motor complications resulted in a decrease in the strength of the correlation between multiple sleep disorders and quality of life. Motor complications had a statistically significant indirect effect on the relationship between multiple sleep disorders and quality of life. Although the size of the indirect effect was moderate, despite worse sleep quality, a patient may experience a further improvement in quality of life when his or her motor complications are treated, and motor function is better maintained.

The strengths of this study include the systematic assessment of sleep disorders in a relatively sizeable multicenter sample of patients with PD. There are two implications for the present findings. First, it may be valuable for clinicians to consider how multiple comorbid sleep disorders relate to quality of life in people with PD. Further assessment could provide a clearer understanding of these relationships, enabling the design and implementation of comprehensive therapeutic approaches. Second, the present findings highlight the complex mediating effect between multiple comorbid sleep disorders and motor complications. Previous research has demonstrated that PD patients with motor complications are more likely to suffer from a lower quality of life^21^. Revealing the impact of motor complications and impaired sleep problems may help clinicians better manage symptoms to improve the quality of life.

Nonetheless, this study also has several limitations. First, our study was a cross-sectional study and did not include premorbid or de novo PD patients. Therefore, the causality between multiple comorbid sleep disorders, motor complications, and quality of life cannot be confirmed. It is crucial to perform prospective studies to investigate causality. Second, an increasing number of sleep disorder screening tools have been developed for use in research and clinical practice. Therefore, cross-comparison between different cohorts is often limited by the types of screening tool used. Finally, assessments of motor complications were limited to the UPDRS IV rather than the scale for wearing off and dyskinesia. Hence, the detailed characteristics of motor complications in patients could not be identified.

In summary, our large-scale, cross-sectional study indicates a close relationship between multiple comorbid sleep disorders and reduced quality of life in PD patients. In addition, motor complications may at least in part mediate the detrimental effects on quality of life caused by multiple comorbid sleep disorders. Therefore, complete and comprehensive assessments of all types of sleep disorders and motor complications in PD patients is imperative to improve the quality of life.

## Methods

### Study design and participants

We performed a multicenter, observational, outpatient-based, cross-sectional study entitled, “Nocturnal symptoms and quality of life in patients with Parkinson’s disease in Shanghai” (SHAPD, clinical trail.gov ID: NCT04023201). From June to November of 2019, the study recruited 1006 PD patients from the clinics of 40 hospitals in Shanghai. Patients had been diagnosed with PD according to the Movement Disorder Society (MDS) PD Criteria^22^. Patients with secondary Parkinsonism, stroke, brain tumor, or an alternative cause for parkinsonism symptoms were excluded. Written informed consent was obtained from all participants, and the study was performed with the approval of the Ethics Committee of Xinhua Hospital affiliated to the Shanghai Jiao Tong University School of Medicine and the Research Ethics Committee of each site in the SHAPD study group.

### Clinical assessment

All patients received standardized assessments. The assessments included an evaluation of demographic and clinical characteristics, disease duration, family history of PD, and medication history for PD-related complications. Total LEDD was calculated according to a previously suggested conversion formulae^23^. There were in total 45 examiners from all 40 study sites who attended training and calibration sessions to prevent inter-rater variability before recruitment. All PD subjects were on medication for their condition at the time of evaluation. The motor and non-motor symptoms of PD were measured with the following scales: the UPDRS, the modified HY stage, Mini-Mental State Examination (MMSE), and the NMSS. Motor fluctuation scores (UPDRS questions 36–39) and dyskinesia scores (32–34) were summed to assess the severity of motor complications related to dopaminergic therapy.

The following instruments were administered by qualified examiners. Nocturnal sleep impairments were assessed using the PD Sleep Scale-2, an instrument that was appraised as “recommended” by the MDS Sleep Scale Task Force^16^. Patients who report complaints of nighttime problems are considered to be suffering from nighttime problems if they score 15 or more^24^. EDS was screened using the Epworth Sleepiness Scale. Patients are considered to be suffering from EDS if they score 10 or more^25^. RBD was screened using the Rapid Eye Movement Sleep Behavior Disorder Questionnaire-Hong Kong. Patients are considered to be suffering from probable RBD (pRBD) if they score 19 or more^26^. An RLS diagnosis was based on questionnaire-based screening. In agreement with the International RLS Study Group criteria for epidemiology studies^27^, participants were considered to have RLS if they reported unpleasant leg sensations combined with an urge to move their legs, occurring mainly at rest, improving with movement, and worsening in the evening or night. The International Restless Legs Scale was used to assess RLS severity^28^.

### Statistical analysis

Summary statistics (means, SDs, and frequencies) were computed for all variables of interest. Between-group differences were determined by *t*-tests (continuous variables) or *χ*^2^ tests (categorical variables). We performed logistic regression analysis for nighttime problems, EDS, pRBD, or RLS as dependent variables to analyze risk factors that could be independently associated with each sleep disorder. The linear trends in clinical characteristics, according to an increased number of sleep disorders, were tested by linear regression analysis for continuous variables and logistic regression analysis for proportional variables. Unstandardized regression coefficients (*β*s) and 95% CIs were calculated for motor complications and the quality of life associated with the number of sleep disorders using multivariable-adjusted models. Linear regression models were adjusted for sex, age, disease duration, PD severity, and dopaminergic therapy. We performed tests for linear trends by entering the number of sleep disorders (0–4) as a continuous variable in the models.

Mediation analyses were conducted with SPSS mediation modeling software and PROCESS^29^ to evaluate whether the severity of motor complications mediated the associations between multiple comorbid sleep disorders and the quality of life. The analysis considered the total effect of multiple sleep disorders (number of sleep disorders) on the quality of life (PDQ-39) and the indirect effect mediated by the motor complications score. A bootstrap estimation approach with 5000 samples was used to measure the indirect effect^30^ with 95% CIs. The indirect effect was considered significant when the 95% CIs did not contain zero^31^.

Statistical significance was set at *P* < 0.05. Statistical computations were performed by SPSS Statistics 26.0 (IBM Corp., Armonk, New York, USA), and OriginPro 2019b software (OriginLab Corp., Northampton, Massachusetts, USA) was used to generate the figure.

### Reporting summary

Further information on research design is available in the Nature Research Reporting Summary linked to this article.

## Supplementary information

SUPPLEMENTAL TABLE

Reporting Summary Checklist FLAT

## Data Availability

The data are available from the corresponding author upon reasonable request.

## Code availability

Analytic code can be made available upon request to the corresponding author.
