# Supplementary material for: Multiple comorbid sleep disorders adversely affect quality of life in Parkinson’s disease patients
Source: NPJ Parkinsons Dis. 2020 Sep 15;6:25. doi: 10.1038/s41531-020-00126-x (PMC7492275; doi:10.1038/s41531-020-00126-x)
Supplement: Supplementary file 1 — SUPPLEMENTAL TABLE [file 41531_2020_126_MOESM1_ESM.pdf]

Supplementary Table 1 Logistic regression analysis of different types of sleep disorders

	OR	95% CI	*P* Value
Insomnia			
UPDRS part IV (32-39)	1.186	1.118, 1.259	<0.001
NMSS score	1.030	1.024, 1.035	<0.001
EDS			
UPDRS part IV (32-39)	1.090	1.035, 1.147	0.001
NMSS score	1.018	1.014, 1.022	<0.001
Hallucination	1.595	1.016, 2.504	0.043
RBD			
Age	0.983	0.968, 0.999	0.038
NMSS score	1.014	1.010, 1.018	<0.001
Hallucination	2.367	1.513, 3.703	<0.001
RLS			
Age	0.979	0.963, 0.997	0.019
Disease duration	1.045	1.010, 1.081	0.012
Modified HY stage	0.723	0.571, 0.914	0.007
UPDRS part IV (32-39)	1.182	1.115, 1.252	<0.001
UPDRS part Ⅱ	1.062	1.036, 1.088	<0.001
DAs medication	0.571	0.429, 0.761	<0.001

Dependent variables: Insomnia, EDS, RBD, RLS; Independent variables: Sex, Age, Disease duration, Modified HY stage, UPDRS parts Ⅱ, Ⅲ and Ⅳ, NMSS score, MMSE score, L-dopa medication, DAs medication, Levodopa equivalent daily dose, Hallucination. Using a likelihood ratio forward selection.

Abbreviations: EDS, excessive daytime sleepiness; RBD, Rapid eye movement sleep behavior disorder; RLS, Restless legs syndrome; Modified HY stage, Modified Hoehn and Yahr stage; UPDRS, Unified Parkinson’s Disease Rating Scale; NMSS, Non-Motor Symptom Scale; MMSE, Mini-Mental State Examination; DA, Dopamine agonist

Supplementary Table 2. Shanghai Parkinson's disease Study Group(SHAP) Members

PI	Affiliation	Email
Zhenguo Liu	Department of Neurology, Xinhua Hospital, Affiliated to Shanghai JiaoTong University, School of Medicine，Shanghai, China	liuzhenguo@xinhuamed.com.cn
Wentao Li	Department of neurology, Shanghai municipal Hospital of Traditional Chinese Medicine，Shanghai University of Traditional Chinese Medicine ,Shanghai， China	lwt1132@163.com
Dongya Huang	Department of Neurology， East Hospital， Tongji University School of Medicine， Shanghai 200120， China	dongyahuang77@hotmail.com
Wei Chen	Department of Neurology, Shanghai Ninth People’s Hospital, Shanghai Jiao Tong University School of Medicine，Shanghai, China	david_chen8106@hotmail.com,
Canxing Yuan	Department of Neurology, Longhua Hospital Shanghai University of Traditional Chinese Medicine， Shanghai, China	18917763103@163.com
Lirong Jin	Department of Neurology，Zhongshan hospital，Fudan University ， Shanghai, China	jinlr99@163.com
Yuhui Wang	Department of Neurology, Shanghai Punan Hospital， Shanghai, China	yhwangch@sina.com
Lingjing Jin	Department of Neurology, Tongji Hospital, Tongji University school of Medicine， Shanghai, China	lingjingjin@163.com
Lu Lei	Department of neurology, Shanghai Jiao Tong University Affiliated Sixth People's Hospital, No.600, Yishan Road, Shanghai, China	lulei@hotmail.com
Xiaoping Wang	Department of Neurology, TongRen Hospital, Shanghai Jiao Tong University School of Medicine, Shanghai, China	x_p_wang@sjtu.edu.cn
Changde Wang	Department of neurology, Shanghai TCM-Integrated Hospital Affiliated to Shanghai University of TCM, Shanghai, China	wangch_de@163.com
Xiaohui Zhao	Department of Neurology, Shanghai Pudong New Area People’s Hospital， Shanghai, China	zhaoxh99990@sina.com
Shan Gao	Department of Neurology, Shanghai JiaoTong University affiliated the Sixth People Hospital, South Campus， Shanghai,	gaoshanwater@163.co

	China	m
Yi Zhao	Department of Neurology, Xuhui Center Hospital in Shanghai， Shanghai, China	Luttyzhao@126.com
Lihong Huang	Department of Neurology, Zhabei Center hospital, Jiang’an District， Shanghai, China	brainh@126.com
Yanxin Zhao	Department of Neurology, Tenth People's Hospital Affiliated to Tongji University， Shanghai, China	zhao_yanxin@126.com
Hui Wang	Department of Neurology, Dahua Hospital of Xu hui District， Shanghai, China	wanghui71887722@163.com
Jing Zhao	Department of Neurology, Minhang Center hospital, Fudan University， Shanghai, China	zhaojingssmu@163.com
Feng Wang	Department of Neurology, Seventh People's Hospital of Shanghai University of Traditional Chinese Medicine, Shanghai, China	13816566556@163.com
Chaorong Zhao	Department of TCM , Shanghai Putuo District Hospital of Traditional Chinese Medicine， Shanghai, China	rongrongzcr@qq.com
De Shi	Department of Neurology, Xinhua hospital Affiliated to Shanghai Jiaotong University School of Medicine,Chongming Branch， Shanghai, China	shide1010@163.com
Wenshi Wei	Department of Neurology, Huadong Hospital, Fudan University, Shanghai, China	Wenshiwei1999@163.com
Xiaojun Hou	Department of Neurology, The Navy Medical University affiliated Changhai Hospital， Shanghai, China	13311931663@163.com
Guojun Luo	Department of Neurology, Jinshan Branch of Shanghai Sixth People's Hospital,Shanghai China.	junlgsy@163.com
Wen Li	Department of Neurology, Kong Jiang Hospital of Yang pu district， Shanghai, China	jjjtong67@163.com
Jun Liu	Department of Neurology, Ruijin Hospital, Affiliated to Shanghai Jiaotong University School of Medicine， Shanghai, China	jly0520@163.com
Wenzhao Wang	Department of Neurology, Shanghai Changzheng Hospital， Shanghai, China	wangwenzhao62@sina.com

Jianmin Wang	Department of Neurology, Renhe Hospital, Baoshan District， Shanghai, China	wjmsu27@163.com
Jialan Sun	Department of Neurology, Gongli Hospital, Pu Dong New Area， Shanghai, China	sjlmed@sina.com
Feng Yu	Department of Neurology, Jiangwan hospital, Hong kou district， Shanghai, China	yufeng1301@126.com
Weiwen Wu	Department of Neurology, Zhongshan Hospital Qingpu Branch, Fudan University， Shanghai, China	weiwenwu0609@163.com
Weidong Pan	Department of Neurology, ShuGuang Hospital Affiliated to Shanghai University of Traditional Chinese Medicine，Shanghai, China	panwd@medmail.com.cn
Yong Bi	Department of Neurology, Shanghai Fourth People’s Hospital Affiliated to Tongji University School of Medicine，Shanghai, China	aflame@126.com
Wenhai Xiang	Department of Neurology, Nanxiang Hospital, Jiading District, Shanghai, China	xwh75@163.com
Hui Zeng	Department of Neurology, Shanghai Yangpu Hospital of Traditional Chinese Medicine， Shanghai, China	1670288940@qq.com
Yingchun Zhao	Department of Neurology, Shanghai Songjiang District Central Hospital， Shanghai, China	zhaoyingchun9077@163.com
Yunlan Du	Department of neurology, renji Hospital Affiliated to Shanghai Jiao Tong University School of Medicine, Shanghai, China	yunlandu2013@163.com.cn
Hengbing Zu	Department of neurology, Jinshan Hospital Affiliated to Fudan University, Shanghai, China	hbzyy666@163.com
Qing Dong	Department of Neurology, Renji Hospital, South Campus, Affiliated to Shanghai Jiaotong University, School of Medicine， Shanghai, China	dnqn1969@qq.com
Wenjian Hu	Department of neurology, Shanghai Baoshan Hospital of Integrated Traditional Chinese and Western Medicine, Shanghai, China	726592156@qq.com
