# Supplementary material for: Multiple comorbid sleep disorders adversely affect quality of life in Parkinson’s disease patients
Source: NPJ Parkinsons Dis. 2020 Sep 15;6:25. doi: 10.1038/s41531-020-00126-x (PMC7492275; doi:10.1038/s41531-020-00126-x)
Supplement: Supplementary file 2 — Reporting Summary Checklist FLAT [file 41531_2020_126_MOESM2_ESM.pdf]

nature research

Corresponding author(s): DBPR NPJPARKD-00443

Last updated by author(s): Jul 9, 2020

Reporting Summary

Nature Research wishes to improve the reproducibility of the work that we publish. This form provides structure for consistency and transparency in reporting. For further information on Nature Research policies, see our Editorial Policies and the Editorial Policy Checklist.

Statistics

For all statistical analyses, confirm that the following items are present in the figure legend, table legend, main text, or Methods section.

n/a	Confirmed	
☐	☒	The exact sample size (*n*) for each experimental group/condition, given as a discrete number and unit of measurement
☐	☒	A statement on whether measurements were taken from distinct samples or whether the same sample was measured repeatedly
☐	☒	The statistical test(s) used AND whether they are one- or two-sided<br>*Only common tests should be described solely by name; describe more complex techniques in the Methods section.*
☐	☒	A description of all covariates tested
☐	☒	A description of any assumptions or corrections, such as tests of normality and adjustment for multiple comparisons
☐	☒	A full description of the statistical parameters including central tendency (e.g. means) or other basic estimates (e.g. regression coefficient) AND variation (e.g. standard deviation) or associated estimates of uncertainty (e.g. confidence intervals)
☐	☒	For null hypothesis testing, the test statistic (e.g. F, t, r) with confidence intervals, effect sizes, degrees of freedom and P value noted<br>*Give P values as exact values whenever suitable.*
☒	☐	For Bayesian analysis, information on the choice of priors and Markov chain Monte Carlo settings
☐	☒	For hierarchical and complex designs, identification of the appropriate level for tests and full reporting of outcomes
☐	☒	Estimates of effect sizes (e.g. Cohen's d, Pearson's r), indicating how they were calculated

Our web collection on statistics for biologists contains articles on many of the points above.

Software and code

Policy information about availability of computer code

Data collection	no software was used
Data analysis	SPSS Statistics 26.0 and OriginPro 2019b software

For manuscripts utilizing custom algorithms or software that are central to the research but not yet described in published literature, software must be made available to editors and reviewers. We strongly encourage code deposition in a community repository (e.g. GitHub). See the Nature Research guidelines for submitting code & software for further information.

Data

Policy information about availability of data

All manuscripts must include a data availability statement. This statement should provide the following information, where applicable:

- Accession codes, unique identifiers, or web links for publicly available datasets
- A list of figures that have associated raw data
- A description of any restrictions on data availability

The data are available from the corresponding author upon reasonable request.

Field-specific reporting

Please select the one below that is the best fit for your research. If you are not sure, read the appropriate sections before making your selection.

☒ Life sciences ☐ Behavioural & social sciences ☐ Ecological, evolutionary & environmental sciences

For a reference copy of the document with all sections, see nature.com/documents/nr-reporting-summary-flat.pdf

Life sciences study design

All studies must disclose on these points even when the disclosure is negative.

Sample size	A total of 1,006 patients with PD (mean age=69.95±8.41 years-old, 577 males) were enrolled in the study.
Data exclusions	No data were excluded from the analyses
Replication	This is a multicenter, observational, outpatient-based, cross-sectional study
Randomization	This is a multicenter, observational, outpatient-based, cross-sectional study
Blinding	This is a multicenter, observational, outpatient-based, cross-sectional study

Reporting for specific materials, systems and methods

We require information from authors about some types of materials, experimental systems and methods used in many studies. Here, indicate whether each material, system or method listed is relevant to your study. If you are not sure if a list item applies to your research, read the appropriate section before selecting a response.

Materials & experimental systems

n/a	Involved in the study	
☒	☐	Antibodies
☒	☐	Eukaryotic cell lines
☒	☐	Palaeontology and archaeology
☒	☐	Animals and other organisms
☐	☒	Human research participants
☐	☒	Clinical data
☒	☐	Dual use research of concern

Methods

n/a	Involved in the study	
☒	☐	ChIP-seq
☒	☐	Flow cytometry
☒	☐	MRI-based neuroimaging

Human research participants

Policy information about studies involving human research participants

Population characteristics	A total of 1,006 patients with PD (mean age=69.95±8.41 years-old, 577 males) were enrolled in the study. The mean PD disease duration was 5.54±4.58 years. The mean modified HY stage was 2.17±0.84 (stage 1-1.5, n=296; stage 2-2.5, n=471; stage≥3, n=223). The UPDRS part I, II, III, and IV scores were 3.06±2.82, 12.41±8.44, 25.50±15.51, and 3.20±3.66, respectively.
Recruitment	We performed a multicenter, observational, outpatient-based, cross-sectional study entitled, “Nocturnal symptoms and quality of life in patients with Parkinson's disease in Shanghai” (SHAPD, clinical trail.gov ID: NCT04023201). From June to November of 2019, the study recruited 1006 PD patients from the clinics of 40 hospitals in Shanghai. Patients had been diagnosed with PD according to the Movement Disorder Society PD Criteria.
Ethics oversight	study was performed with the approval of the Ethics Committee of Xinhua Hospital affiliated to the Shanghai Jiao Tong University School of Medicine and the Research Ethics Committee of each site in the SHAPD study group.

Note that full information on the approval of the study protocol must also be provided in the manuscript.

Clinical data

Policy information about clinical studies

All manuscripts should comply with the ICMJE guidelines for publication of clinical research and a completed CONSORT checklist must be included with all submissions.

Clinical trial registration	NCT04023201

Study protocol	The detailed study protocol is described in the method section of the article
Data collection	All patients received standardized assessments. The assessments included an evaluation of demographic and clinical characteristics
Outcomes	45 examiners from all 40 study sites who attended training and calibration sessions to prevent inter-rater variability before recruitment.
